# Potential of Glucagon-Like Peptide-1 (GLP-1) Receptor Agonists in Mitigating Metabolic Side Effects Associated With Clozapine or Olanzapine Therapy: A Literature Review

**DOI:** 10.7759/cureus.109089

**Published:** 2026-05-18

**Authors:** Amir Meftah, Bolaji Yoade, Cade Cowart, Anthony Choi, Olufemi Popoola, Olorunfemi Afe, Sofia Efremoff, Chino Ezema, Bamidele Johnson, Jeffery Lawrence, Tolu Olupona

**Affiliations:** 1 Psychiatry and Behavioral Sciences, One Brooklyn Health, Interfaith Medical Center, Brooklyn, USA; 2 Psychiatry, Interfaith Medical Center, Brooklyn, USA; 3 College of Medicine, American University of Antigua, Coolidge, ATG; 4 College of Medicine, Touro College of Osteopathic Medicine, New York City, USA; 5 Psychiatry and Behavioral Sciences, Interfaith Medical Center, Brooklyn, USA

**Keywords:** clozapine, dulaglutide, exenatide, glp-1 agonist, glucagon-like peptide-1 receptor agonists, liraglutide, metabolic side effects, olanzapine, schizoaffective disorder, schizophrenia

## Abstract

Clozapine and Olanzapine can cause severe metabolic side effects, increasing cardiovascular risk. Glucagon-like peptide-1 (GLP-1) receptor agonists, primarily approved for use in patients with type 2 diabetes, may counter these effects by promoting weight loss, improving glucose metabolism, and enhancing cardiovascular health, offering a promising approach for managing antipsychotic-related metabolic complications. We conducted a literature review using the search terms "clozapine OR olanzapine AND GLP-1 agonist," "schizophrenia AND GLP-1 agonist," "antipsychotic AND GLP-1 agonist," and "psychosis AND GLP-1 agonist" on PubMed and Google Scholar. This search initially yielded 172 articles. We focused on clinical trials or case reports investigating the use of GLP-1 agonists in conjunction with Olanzapine or Clozapine. Our final selection included two clinical trial studies, one case report, and a retrospective study. In a case report, a schizophrenic patient on Clozapine treated with liraglutide experienced a reduction in glycated hemoglobin (HbA1c ), from 10% to 6.5% over 14 months, along with a total weight loss of 7.7 kg (8.7% body weight reduction) after two years, and decreased insulin requirements. In a retrospective study reviewing 16 obese patients with schizophrenia treated with liraglutide, of whom 14 were on Clozapine, significant reductions in body weight, waist circumference, body mass index (BMI), and plasma glucose levels were observed over 16 weeks (*P* < 0.001). A randomized clinical trial with 103 patients, with diagnoses within the schizophrenia spectrum disorders, who have been treated with Olanzapine or Clozapine, and liraglutide, showed significant improvement in glucose tolerance when compared to placebo (*P* < 0.001), with 63.8% of liraglutide-treated patients achieving normal glucose tolerance versus 16.0% in the placebo group. Additional benefits included significant reductions in body weight, waist circumference, blood pressure, visceral fat, and low-density lipoprotein (LDL) cholesterol levels. In another outpatient study with 28 participants randomized to receive subcutaneous, extended-release exenatide in one arm, or standard of care in the other arm for 24 weeks, six participants in the exenatide group achieved more than 5% weight loss, compared to just one in the usual care group (*P* = 0.029). The exenatide group experienced significantly greater mean weight loss compared to the usual care group (*P* = 0.015). Additionally, the exenatide group showed a more substantial reduction in BMI (*P* = 0.019) and improved glycemic control, with significant reductions in fasting glucose (*P* = 0.036) and glycated hemoglobin (*P* = 0.004) compared to the usual care group. This review underscores the need for future studies that involve the use of GLP-1 agonists in conjunction with Clozapine or Olanzapine. Although results from preliminary studies are promising, there is a need for more randomized controlled clinical trials to validate these findings, assess long-term efficacy, and develop clinical guidelines.

## Introduction and background

Clozapine and Olanzapine are second-generation antipsychotics widely recognized for their efficacy in treating various psychotic disorders, including schizophrenia, schizoaffective disorder, and bipolar mania [1-4]. Their preferential use over first-generation antipsychotics is largely attributed to a lower risk of extrapyramidal symptoms [5]. However, their therapeutic benefits are often overshadowed by notable metabolic complications, including weight gain, resistance to insulin, dyslipidemia, and a higher likelihood of developing metabolic syndrome [5,6]. Although the mechanisms by which Clozapine and Olanzapine lead to these metabolic complications are not fully understood, antipsychotic blockage of serotonin (5HT2C) and histamine (H1) receptors in the brain has been implicated in increased food cravings and insulin resistance - leading to a slowing of metabolism and weight gain [6]. Approximately 40%-60% of patients treated with Clozapine or Olanzapine develop clinically significant weight gain, and up to one-third may meet criteria for metabolic syndrome within one year of treatment initiation [7,8]. These adverse effects not only contribute to poor medication adherence but also elevate the long-term risk of cardiovascular disease and premature mortality, posing a major challenge in clinical practice [7,9].

Non-pharmacologic interventions, such as lifestyle and dietary modifications, have shown limited effectiveness in counteracting these metabolic disturbances. This may be because, while antipsychotic-induced weight gain plays a significant role in the development of glucose intolerance, there is still a considerable group of individuals who develop insulin insensitivity without the prospective weight gain. Studies on rat models have shown a significant dose-dependent decrease in insulin sensitivity 2 hours following the administration of Clozapine and Olanzapine [9,10]. As a result, there is growing interest in identifying adjunctive pharmacological strategies that can attenuate or reverse the metabolic consequences of antipsychotic therapy.

Glucagon-like peptide-1 (GLP-1) receptor agonists (RAs) were initially produced to manage type two diabetes mellitus, but they have also shown significant potential in supporting weight reduction, improving insulin sensitivity, and enhancing overall cardiometabolic health [11]. Emerging research suggests that these agents may also counteract the adverse metabolic effects associated with antipsychotic medications, particularly Clozapine and Olanzapine [12,13]. GLP-1 RAs enhance the release of insulin and prevent the release of glucagon, thereby limiting the post-meal increase in blood glucose [14]. Their mechanism of action involves central appetite regulation and peripheral metabolic modulation, making them promising candidates for integration into psychiatric pharmacotherapy [13,14].

This review aims to summarize existing clinical as well as experimental findings on the use of GLP-1 RAs as adjunctive treatments in patients receiving Clozapine or Olanzapine, with a focus on outcomes related to weight gain, metabolic syndrome, and cardiovascular risk.

## Review

Historical use of Clozapine and Olanzapine

The development of antipsychotic medications has been a cornerstone in the management of patients with schizophrenia as well as other psychotic disorders. Among these, Clozapine and Olanzapine have stood out due to their unique efficacy and pharmacological profiles [1,3].

Emergence of Clozapine

Clozapine was first synthesized in 1958 by researchers at the Swiss company Wander AG [15,16]. Initially, it was developed as part of a class of tricyclic compounds structurally related to imipramine [17]. It was introduced in Europe in the early 1970s and marketed as a novel antipsychotic with fewer extrapyramidal symptoms (EPS) than first-generation agents [15,16]. Unlike traditional dopamine D2 antagonists, Clozapine displayed weak D2 receptor affinity but strong serotonergic (5-HT2A), adrenergic, histaminergic, and muscarinic antagonism [18]. This receptor profile underlies its superior efficacy and lower propensity to cause EPS [19]. Despite its clinical potential, Clozapine was withdrawn in 1975 in many countries due to reports of agranulocytosis, a potentially fatal drop in white blood cells [16,20]. However, its reemergence was prompted by a pivotal U.S. study, “Clozapine for the treatment-resistant schizophrenic: A double-blind comparison with chlorpromazine” in 1988, which demonstrated Clozapine's superiority in treatment-resistant schizophrenia [17,21]. This landmark study, a randomized double-blind trial, showed that Clozapine was effective in approximately 30% of patients unresponsive to conventional neuroleptics [21]. Based on these findings, the U.S. FDA approved Clozapine in 1990 for treatment-resistant schizophrenia, but only under strict hematological monitoring [4,22].

Clozapine also demonstrated efficacy in reducing suicide risk among patients with schizophrenia, a finding later confirmed in the InterSePT trial [23]. As a result, in 2002, the FDA expanded its indications to include the reduction of suicidal behavior in schizophrenia and schizoaffective disorder [23]. Despite its unparalleled efficacy, Clozapine remains underutilized due to monitoring burdens, clinician hesitancy, and concerns over side effects such as metabolic syndrome, myocarditis, and sialorrhea [17,24-26].

Development of Olanzapine

Olanzapine was developed in the 1990s by Eli Lilly as part of an effort to create an antipsychotic with Clozapine-like efficacy but fewer adverse effects [27,28]. It was approved by the FDA in 1996 for the treatment of schizophrenia and later for bipolar disorder [1]. Chemically, Olanzapine is a thienobenzodiazepine, structurally related to Clozapine, and shares a similar receptor binding profile, including potent antagonism at 5-HT2A, D2, H1, M1, and alpha-1 adrenergic receptors [1]. Clinical trials demonstrated Olanzapine's efficacy in treating the negative and positive manifestations of schizophrenia, while posing a lower risk of extrapyramidal complications than haloperidol and other first-generation agents [27,29]. Of the second-generation antipsychotics, Olanzapine became one of the most frequently prescribed in the late 1990's as well as in the early 2000's [5,30]. Albeit its favorable profile in the management of acute agitation, relapse prevention, and bipolar mania, which contributed to its popularity in psychiatric practice [27,31], widespread use revealed significant concerns regarding Olanzapine's metabolic side effects. These include substantial weight gain, abnormal lipid profile, reduced insulin sensitivity, and a higher likelihood of type two diabetes [24,32].

In head-to-head trials such as the CATIE study, Olanzapine was identified as one of the most beneficial antipsychotics in managing symptoms, but it was also associated with the highest rates of metabolic adverse effects [30]. Due to these risks, guidelines now recommend careful patient selection and metabolic monitoring when prescribing Olanzapine, especially in populations vulnerable to metabolic disease [24,33].

Comparative considerations

Both Clozapine and Olanzapine belong to the second-generation (atypical) antipsychotic class, yet they occupy different clinical niches. Clozapine remains the gold standard for treatment-resistant schizophrenia and suicidality [16,19], while Olanzapine is considered a first-line option for acute psychosis and mood episodes, albeit with metabolic caveats [27,34]. Notably, both agents exert antipsychotic effects with relatively lower D2 receptor occupancy compared to first-generation antipsychotics, a feature thought to underlie their reduced EPS liability [10,35]. Their high affinity for serotonin receptors, particularly 5-HT2A antagonism, has been proposed to enhance dopamine release in nigrostriatal pathways, mitigating motor side effects [19,35]. However, both Clozapine and Olanzapine have strong histamine H1 and muscarinic M3 antagonism, contributing to sedation, weight gain, and metabolic disturbances [10,28].

Clozapine’s unique immunological side effects (e.g., agranulocytosis, eosinophilia, myocarditis) distinguish it from other antipsychotics and necessitate specialized prescribing practices and, until recently, registry enrollment [22,36]. In contrast, concerns with Olanzapine’s safety profile are largely metabolic and cardiovascular. Although less acutely dangerous, these can lead to long-term morbidity and mortality [8].

Evolution in clinical practice

The increasing clinical use of these two drugs reflects a broader shift in psychiatry from managing psychosis with sedatives and typical antipsychotics toward more targeted and effective pharmacotherapy. Clozapine, once nearly abandoned due to safety concerns, is now regarded as the most effective antipsychotic for refractory cases [16,37]. Efforts are ongoing to reduce barriers to its use, including point-of-care neutrophil monitoring and educational campaigns for prescribers [22,26]. Olanzapine, meanwhile, is a case study in the trade-offs inherent in psychopharmacology: its efficacy is among the highest of non-Clozapine antipsychotic agents, but its metabolic burden necessitates caution [6,34]. Albeit recent formulations like Olanzapine-samidorphan (an opioid antagonist combination) have been found to mitigate medication-induced weight gain, while preserving efficacy, it does not necessarily reduce weight gained before the onset of use [38]. 

Nevertheless, together, Clozapine and Olanzapine exemplify how psychiatric therapeutics evolve by balancing efficacy, tolerability, and safety in diverse patient populations.

History of GLP-1 usage

The therapeutic history of GLP-1 spans 3 decades and began with its discovery as an incretin peptide produced by the L-cells of the gastrointestinal tract in response to food ingestion [14,39]. GLP-1 was identified in the 1980s as part of the proglucagon peptide family and shown to enhance insulin secretion in a glucose-dependent manner [40]. Findings indicated that the cleavage of a large proglucagon GLP-1 into smaller active GLP-1 peptides was more efficient in the gut when compared to the pancreas and further supported the incretin hypothesis [14,39]. Researchers discovered that GLP-1 has multiple physiological actions, including slowing gastric emptying, reducing glucagon secretion, and promoting satiety, making it a promising candidate for diabetes treatment [14,41]. Nevertheless, native GLP-1 exhibits a brief half-life (approximately one to two minutes) as a result of quick enzymatic breakdown by dipeptidyl peptidase-4 (DPP-4) [42,43]. The development of GLP-1 RAs, which mimic the natural hormone but resist DPP-4 degradation, has helped to extend their half-life and therapeutic efficacy [43]. The first such agent, exenatide, was approved by the FDA in 2005 for type 2 diabetes [44]. It was derived from exendin-4, a peptide found in Gila monster saliva, which shares homology with GLP-1 [45]. Soon after, longer-acting agents such as liraglutide (once daily) and dulaglutide (once weekly) entered the market, offering better glycemic control and convenience [46]. More recently, new drugs such as semaglutide and tirzepatide have demonstrated even better efficacy [47,48].

Beyond glycemic benefits, GLP-1 RAs showed remarkable effects on body weight, which attracted attention to their use in obesity management [49]. In 2014, liraglutide was FDA-approved under a higher dose (3.0 mg) specifically for weight management, marketed as Saxenda [50]. GLP-1 agonists also showed cardiovascular benefits, as demonstrated by large, randomized trials such as LEADER (liraglutide) and REWIND (dulaglutide), leading to their FDA approval for reducing cardiovascular risk in patients with diabetes and established cardiovascular disease [51,52]. More recently, semaglutide, a once-weekly GLP-1 RA, has redefined metabolic therapy. It was first approved as Ozempic for diabetes in 2017, and later as Wegovy for obesity in 2021, based on STEP trials showing 15-20% weight loss [11,48].

In psychiatry, interest in GLP-1 RAs has emerged and is increasing due to their potential in mitigating antipsychotic-induced weight gain and insulin resistance, particularly with agents like Clozapine and Olanzapine [53]. These effects may be due in part to reduced appetite and food intake via central hypothalamic and mesolimbic pathways, thereby implicating GLP-1 receptors in reward and motivation systems, as shown in preclinical studies [39,54].

The safety profiles, side effects, and indications for usage of Olanzapine, Clozapine, and GLP-1 RAs can be found in Table 1.

**Table 1:** Safety profiles and side effects: Olanzapine, Clozapine, and GLP-1 RAs; overview of known and reported side-effect profiles of Olanzapine, Clozapine, and GLP-1 RAs as well as indications of use. GLP-1 RAs, glucagon-like peptide-1 receptor agonists; CV, cardiovascular

Category	Olanzapine	Clozapine	GLP-1 RAs
Metabolic Effects	High (weight gain, hyperglycemia, dyslipidemia, and increased risk for type 2 diabetes, high metabolic CV risk) [8,30]	Very high (severe weight gain, hyperglycemia, and dyslipidemia) [55]	Beneficial (weight loss, improved glucose) [46,49]
Anticholinergic	Moderate to high (dry mouth) [27]	Constipation [56]	Anticholinergic side effects are not characterized in reported side effect profiles [57]
Antihistaminergic effect	Moderate to high (sedation) [27]	High (sedation) [5]	None reported [57]
Agranulocytosis	Rare [58]	Yes (~1%) [59]	None reported [60]
GI effects	Moderate (constipation) [27]	Severe (Ileus, Bowel necrosis) [61]	Moderate (dose-dependent nausea, vomiting, diarrhea, and abdominal discomfort) [62]; moderate (delayed gastric emptying and central appetite suppression) [43]
Seizure risk	Low [63]	High (dose-dependent) [64]	
CV risk	Moderate (mild QTc prolongation) [65]; moderate (orthostatic hypotension) [28]	High (tachycardia, ECG abnormalities, orthostatic hypotension) [24]; high (myocarditis, CV mortality risk) [25]	Beneficial cardioprotective [52]
Hypoglycemia	Rare cases with insulin hypersecretion [66]	Rare [67]	Rare [68]
Pancreatitis	Rare [69]	Rare complication following termination of treatment [70]	Low [71]
Thyroid C-cell tumors (in rodents)	Not characterized within the side effect profile	Not characterized within the side effect profile	Contraindicated in MEN 2 or medullary thyroid carcinoma family history [11]
Psychiatric indications	First-line for schizophrenia and bipolar [34]	For treatment-resistant schizophrenia and suicidality [21,23]	Investigation for antipsychotic Induced weight gain [53]

Material and methods

We conducted a literature search to identify studies involving individuals with schizophrenia or related psychotic disorders treated with Clozapine or Olanzapine, and receiving adjunctive treatment with GLP-1 RAs. Searches were performed in PubMed and Google Scholar up to September 2024. The following search terms were used in various combinations: “clozapine OR olanzapine AND GLP-1 agonist,” “schizophrenia AND GLP-1 agonist,” “antipsychotic AND GLP-1 agonist,” and “psychosis AND GLP-1 agonist.” The initial search yielded 172 articles. Titles and abstracts were screened to exclude review articles and studies that examined GLP-1 agonists in psychiatric populations not receiving Clozapine or Olanzapine. We then focused on clinical studies that directly investigated the adjunctive use of GLP-1 agonists in patients receiving Clozapine or Olanzapine. After full-text review, two randomized clinical trials, one case report, and one retrospective study were identified and included in the final analysis. These studies were reviewed and synthesized to produce an overview of existing data on the safety, tolerability, and therapeutic efficacy of the agonists of GLP-1 receptors in this population.

Discussion

The following is a brief, comprehensive analysis of the endpoint data discussed in each study of this review.

Larsen et al. conducted a randomized clinical double-blind trial utilizing 103 participants with a diagnosis within the schizophrenia spectrum and treated with Olanzapine or Clozapine [12]. Participants also had both prediabetes and obesity. The participants were randomly placed into two groups and treated for 16-weeks: one receiving treatment with subcutaneous liraglutide (*n* = 47) and one receiving treatment with a prefilled pen placebo (*n* = 50) [12]. The authors reported that the liraglutide treatment group saw significant changes in body weight, reduction in waist circumference, body mass index (BMI), improved glucose tolerance, reduction in visceral fat, as well as a reduction of approximately 15.4 mg/dL in the levels of low-density lipoprotein when compared to the placebo group [12].

Lee et al. conducted a retrospective study utilizing 16 individuals with obesity and a diagnosis of schizophrenia or bipolar disorder being treated with Olanzapine or Clozapine [72]. The study examined 16 weeks of treatment with liraglutide 3.0 mg and measured body weight changes as well as the Clinical Global Impression-Severity scale. Participants were then divided into responders and non-responders, classified as a loss of at least 5% or more of body fat [72]. The authors reported that amongst patients treated for schizophrenia and bipolar disorder with Clozapine in addition to liraglutide 3 mg/day, a significant reduction in body weight was observed in 16 obese patients. The study indicated that there was also a significant improvement in associated cardiometabolic risk factors like waist circumference, BMI, and plasma glucose levels [72].

The use of exenatide 2 mg/weekly subcutaneous injection as an adjunctive treatment with Clozapine for schizophrenia was reported by Siskind et al. at 24 weeks, six participants treated with Exenatide achieved a weight loss of more than 5%, whereas only 1 participant in the usual care group experienced a weight loss of greater than 5% [73]. Participants treated with Exenatide also demonstrated significant weight loss, including reductions in BMI, waist circumference, HbA1c, and fasting glucose levels [73].

Ishøy et al. indicated a reduction in baseline weight of 5.1 kg at three months of treatment and then 7.7 kg at two years of treatment with the use of liraglutide as an adjunctive treatment to Clozapine [74]. An 8.7% total weight reduction was observed [74]. Also, there was an improvement in baseline HbA1C and a reduction in daily insulin usage [74].

Our review was restricted by a lack of data on long-term cardiometabolic effects of GLP-1 agonists in adjunctive treatment with Olanzapine and Clozapine. Additional limitations included the use of small sample sizes that limited power, a lack of a control group due to study designs, and restricted criteria on participation, such as the exclusion of patients with bipolar disorder and patients on polypharmacy. Additional data from randomized clinical trials and post-market surveillance studies can be useful in understanding the effectiveness and safety of individuals on Clozapine and Olanzapine being treated with GLP-1 RAs, to mitigate the cardiometabolic side effects in a diverse population.

A summarized review of the study types, population characteristics, interventions, and results can be found in Table 2.

**Table 2:** Description of included studies, interventions, and reported comparative results. GLP-1, glucagon-like peptide-1; T2DM, type 2 diabetes mellitus

Study	Type of study	Population	Intervention	Baseline to endpoint results
Larsen et al. (2017) [12]	Randomized clinical trial	*N* = 103 with both obesity and prediabetes; randomized to GLP-1 agonist (*n* = 47) treatment or placebo (*n* = 50) [12]	Antipsychotic: Clozapine, Olanzapine, GLP-1: liraglutide [12]	As measured at the 16-week endpoint, 30 participants treated with liraglutide (63.8%) developed normal glucose tolerance, compared with 8 participants treated with placebo (16.0%) (*P* < 0.001; number needed to treat, 2). There was a decrease in body weight in participants who received liraglutide in comparison to those who received placebo (-5.3 kg; 95% confidence interval (CI), -7.0 to -3.7 kg) [12]. Significant reductions were observed in visceral fat (-250.19 g; 95% CI, -459.9 to -40.5 g), systolic blood pressure (-4.9 mmHg; 95% CI, -9.5 to -0.3 mmHg), waist circumference (-4.1 cm; 95% CI, -6.0 to -2.3 cm), and low-density lipoprotein (LDL) levels (-15.4 mg/dL; 95% CI, -23.2 to -7.7 mg/dL), in participants who received liraglutide in comparison to placebo [12]. Significantly, improvement was observed in glucose tolerance, body weight, and cardio-metabolic indices, amongst schizophrenic patients who received liraglutide while undergoing treatments with Olanzapine and Clozapine [12].
Lee et al. (2021) [72]	Retrospective	*N* = 16; obese with diagnosis of schizophrenia or bipolar disorder treated with GLP-1 receptor agonist [72]	Antipsychotic: 14 of 16 on Clozapine, GLP-1: liraglutide 3 mg daily [72].	At the 16-week endpoint, participants who received 3.0 mg developed a significant reduction in body weight, with an estimated marginal mean of 93.2 kg at baseline, and 88.9 kg on completion of 16 weeks of treatment (*P* < 0.001) with reductions in waist circumference, BMI, and plasma glucose levels. Significant reductions were observed in obese patients who received antipsychotics while on treatment with liraglutide [72].
Siskind et al. (2017) [73]	Randomized clinical trial	*N* = 28 (*n* =14 control, *n* =14 intervention) with a diagnosis of schizophrenia or schizoaffective disorder and had been taking Clozapine for at least 18 weeks and had a recorded BMI of 30-45 kg/m^2^. 3/14 in the exenatide group and 2/14 in the usual care group had T2DM [73].	Antipsychotic: Clozapine, GLP-1: Exenatide administered once a week, subcutaneously or usual care, delivered for a duration of 24 weeks [73].	As measured at 12- and 24-week endpoints, six participants who received exenatide developed >5% weight loss vs. one participant who was treated with usual care (*P* = 0.29) [73]. In comparison to participants receiving usual care, those who received exenatide developed greater loss in mean body weight (-5.29 vs. -1.12 kg; *P* = 0.015), with similarly higher reductions in body mass index (-1.78 vs. -0.39 kg/m^2^; *P* = 0.019), glycated hemoglobin levels (-0.21% vs. 0.03%; *P* = 0.04) and fasting glucose levels (-0.34 vs. 0.39 mmol/L; *P* = 0.36) [73]. No significant differences were observed in other components of metabolic syndrome [73].
Ishøy et al. (2013) [74]	Case report	*N* = 1; single patient with a diagnosis of schizophrenia treated with Clozapine and poorly controlled type 2 diabetes, treated with GLP-1 agonist [74].	Antipsychotic: Clozapine, GLP-1: liraglutide 0.6 mg/day at initiation, 1.2 mg/day at three weeks, and 1.8 mg/day at eight months [74].	Following three months of treatment, the participant's HbA1c level reduced to 8.9% and her body weight reduced by 5.1 kg. At the conclusion of two years of treatment, the patient lost 7.7 kg (an 8.7% reduction in body weight) [74]. At the end of 14 months, there was a 6.5 % reduction in participants’ HbA1c level with resultant, gradual decrease in daily insulin needs (28 IU/day) [74].

## Conclusions

Our review highlights the role of GLP-1 RAs in mitigating the metabolic side effects commonly associated with Olanzapine and Clozapine. Adjunctive use of GLP-1 RAs may reduce weight gain, waist circumference, BMI, HbA1c, and fasting serum glucose levels; however, there is a need for long-term clinical data on the use of these agents as adjuncts to Clozapine and Olanzapine to better understand their significance in mitigating their metabolic side effects. Nevertheless, the findings in the reviewed studies have useful clinical implications; improved metabolic outcomes may lead to better tolerability and long-term adherence to Clozapine and Olanzapine treatment, particularly in patients at elevated risk of cardiovascular disease or metabolic syndrome. For the continued use of these medications to be translated into routine clinical practice, further research must focus on the long-term effects of GLP-1 agonists in these contexts, in order to explore additional benefits and possible risks that may be linked to long-term use.
